# Selecting control genes for RT-QPCR using public microarray data

**DOI:** 10.1186/1471-2105-10-42

**Published:** 2009-02-02

**Authors:** Vlad Popovici, Darlene R Goldstein, Janine Antonov, Rolf Jaggi, Mauro Delorenzi, Pratyaksha Wirapati

**Affiliations:** 1 Bioinformatics Core Facility, Swiss Institute of Bioinformatics, CH-1015 Lausanne, Switzerland; 2 Institut de mathématiques (IMA), Ecole Polytechnique Fédérale de Lausanne (EPFL), CH-1015 Lausanne, Switzerland; 3 Department of Clinical Research, University of Bern, CH-3010 Bern, Switzerland

## Abstract

**Background:**

Gene expression analysis has emerged as a major biological research area, with real-time quantitative reverse transcription PCR (RT-QPCR) being one of the most accurate and widely used techniques for expression profiling of selected genes. In order to obtain results that are comparable across assays, a stable normalization strategy is required. In general, the normalization of PCR measurements between different samples uses one to several control genes (*e.g*. housekeeping genes), from which a baseline reference level is constructed. Thus, the choice of the control genes is of utmost importance, yet there is not a generally accepted standard technique for screening a large number of candidates and identifying the best ones.

**Results:**

We propose a novel approach for scoring and ranking candidate genes for their suitability as control genes. Our approach relies on publicly available microarray data and allows the combination of multiple data sets originating from different platforms and/or representing different pathologies. The use of microarray data allows the screening of tens of thousands of genes, producing very comprehensive lists of candidates. We also provide two lists of candidate control genes: one which is breast cancer-specific and one with more general applicability. Two genes from the breast cancer list which had not been previously used as control genes are identified and validated by RT-QPCR. Open source R functions are available at

**Conclusion:**

We proposed a new method for identifying candidate control genes for RT-QPCR which was able to rank thousands of genes according to some predefined suitability criteria and we applied it to the case of breast cancer. We also empirically showed that translating the results from microarray to PCR platform was achievable.

## Background

Real-time quantitative reverse transcription PCR (RT-QPCR) has become a method of choice for gene expression profiling in a large number of applications. However, obtaining reliable measurements still depends on the choice of control genes on which the baseline level is constructed. Selecting the control genes remains a critical point in the normalization process. Often, a short list of candidates is produced based on non-systematic and/or often poorly defined biological considerations.

In early studies, normalization was usually based on a single control gene. More recently, the trend is to use several control genes whose average expression level (on a log-scale) is used as baseline [1,2]. Suitable control genes are selected from a short list of 10–15 genes by ranking them according to a criterion that essentially selects those genes having low variation across samples. We describe brie y a few such methods below.

[2] introduces a stability coefficient which is used along with the coefficient of variation for ranking the genes from a predefined list of candidates. Gene stability is defined in terms of average standard deviation of the log-ratios of pairs of candidate genes. Genes are ranked by iteratively removing those most unstable. This approach has the drawback that repeated comparison of pairs of genes is required, which is feasible only when the number of candidates is small. In addition, the method implicitly assumes that there are no co-regulated genes. A model-based approach proposed by [1] aims at estimating the overall variation as well as the between sample variation of each candidate gene. However, with this approach it is cumbersome to integrate different platforms. In an application to plant pathogen profiling, [3] investigates a list of 18 pre-selected candidate housekeeping genes, using the method proposed in [2] and RT-QPCR for measuring the gene expressions. [4] proposes a PCA-based statistical analysis to identify the most suitable control genes among 13 candidates which were selected such that they had independent functions in cellular maintenance.

[5] introduces a strategy which combines the coefficient of variation, maximum fold change and mean expression value in a ranking criterion that is applied to a large number of samples representing a wide variety of tissues. All these samples were hybridized on either Affymetrix HG-U133A or HG-U133 Plus 2.0 arrays and quantile-normalized together prior to ranking. Only probesets common to both arrays were used, with probesets targeting the same gene averaged into a single value.

There are some important differences between the methods described above and our approach (described below). Firstly, in contrast with all the studies based on PCR, we do not require a short list of candidate genes to be produced before assessing their suitability as control genes. Instead, we screen all the genes represented on the microarray chips, giving us the opportunity to assess genes that have not been reported previously. Moreover, we take a meta-analytical approach to the problem, first creating an independent ranking within each data set then aggregating these rankings into a single list. This approach has the advantage of being platform- and normalization-independent. In addition, the approach is not limited to using only genes common between different data sets. Also, by not using the coefficient of variation, we can treat uniformly both single and two-colors arrays. Thus, we are able to exploit data obtained from different platforms without requiring them to be normalized together. Furthermore, the meta-analytical approach allows us to integrate gene lists produced using our ranking system with other ranked gene lists from the literature and we do not require all data to be normalized together. Another key difference is that we introduce a new stability coefficient that combines the mean expression and the standard deviation in a ranking criterion that corresponds to our requirements for candidate control genes for RT-QPCR. In general, these requirements are:

• low variability across different specimens (*e.g*., subtypes of tumors or normal tissues);

• high and moderate level of expression, such that control genes with expression levels across a larger range may be selected;

• consistency across experiments and platforms.

A key question is whether it is possible to select genes from microarray studies that perform as control genes on PCR platform, given that the two technologies are different. We hypothesize that translating the list of candidate genes from microarray to PCR platform is feasible and we provide empirical evidence in this sense.

## Results

### Data sets and pre-processing steps

We have collected ten publicly available data sets [6-15], listed in Table 1, from which we derived the quantities of interest: the mean and standard deviations of the log-intensities (on Affymetrix platforms) or of the log-ratios (on Agilent platforms).

**Table 1:** The ten public microarray data sets used (*n *= number of samples).

Data set ID and reference	*n*	Platform
BWH [6]	47	Affymetrix U133v2
EMC [7]	286	Affymetrix U133A
EXPO [8]	1375	Affymetrix U133Plus2
JRH2 [9]	61	Affymetrix U133A
MGH [10]	60	Agilent
NKI [11]	337	Agilent (custom)
STOCK [12]	159	Affymetrix U133A, B
TGIF1 [13]	49	Affymetrix U133A
UNC [14]	153	Agilent HuA1
UPP [15]	249	Affymetrix U133A, B

We note here that the original EXPO data set contains a number of different pathologies, but we restrict analysis here to eight different types of cancer (breast, colon, endometrium, kidney, lung, ovary, prostate and uterus) for which a sufficient number of samples existed. EXPO breast cancer samples (*n *= 328) were used to produce both the breast cancer and general cancer lists of candidate genes.

The Affymetrix data are available as MAS5.0 normalized values. The Agilent data contains log-ratios (base 10) and mean-centered log-intensities. The standard deviations of log-intensities (Affymetrix) and log-ratios (Agilent) were used as measures of variability. The means of log-intensities (both Affymetrix and Agilent) were used as measures of average expression level.

When multiple probesets of the same gene are present, only the most variable one is used. We consider all genes from each platform, the aggregation methods used being able to cope with 'missing' genes (those not represented on the array). Considering only those genes common to all platforms is an unnecessary limiting constraint, as increasing the number of data sets and the heterogeneity of the collection leads to a successively smaller intersection of genes.

**Figure 1:**
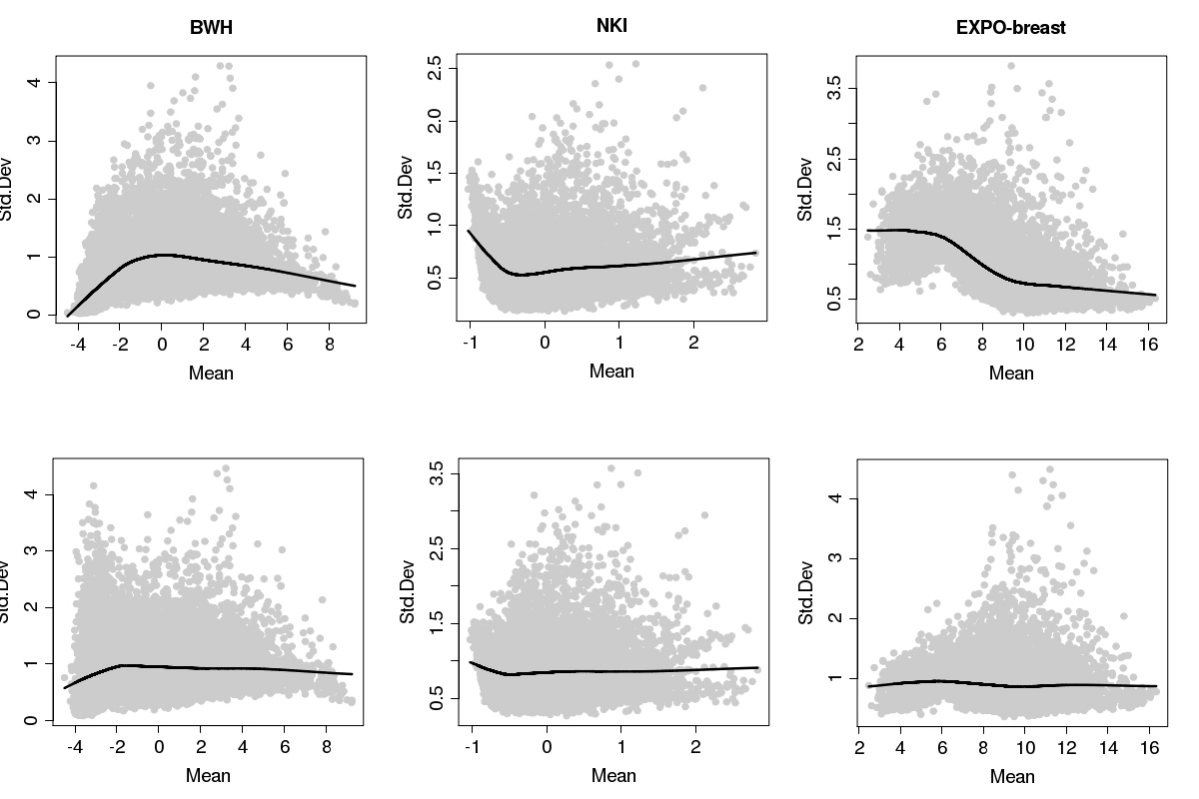
**Example of variance stabilization by LOESS correction**. LOESS correction applied to three data sets: BWH, NKI and EXPO-breast, respectively. The first row shows the original data with the fitted first order LOESS curve, while the second row shows the variance-stabilized data.

Before any further usage of the data, we reduce the variability across platforms by scaling with a factor given by a first order LOESS fit of the data. The effect of this transformation can be seen in Figure 1, where the black line represents the fitted curve. This simple approach seems effective, except for genes with low expression. However, as we are interested in genes with higher mean expression, this deficiency is not problematic.

### Ranking the genes

Let us consider that we have *M *microarray data sets, each containing expression values of a set of genes *G*_*k*_, *k *= 1,...,*M*, and let *G *= ∪_*k*_*G*_*k *_= {1,...,*N*} be the set of all genes represented at least once in any of these data sets.

#### Gene scores

We aim to design a scoring function which ranks the genes such that higher scores correspond to genes that are more suitable to be used as control genes. As mentioned above, the score has to combine each gene's mean expression and standard deviation into a single value such that higher expression levels and lower variances (standard deviations) are favored. Moreover, the score must be independent of the technology used to measure expression levels and the method for normalization.

**Figure 2:**
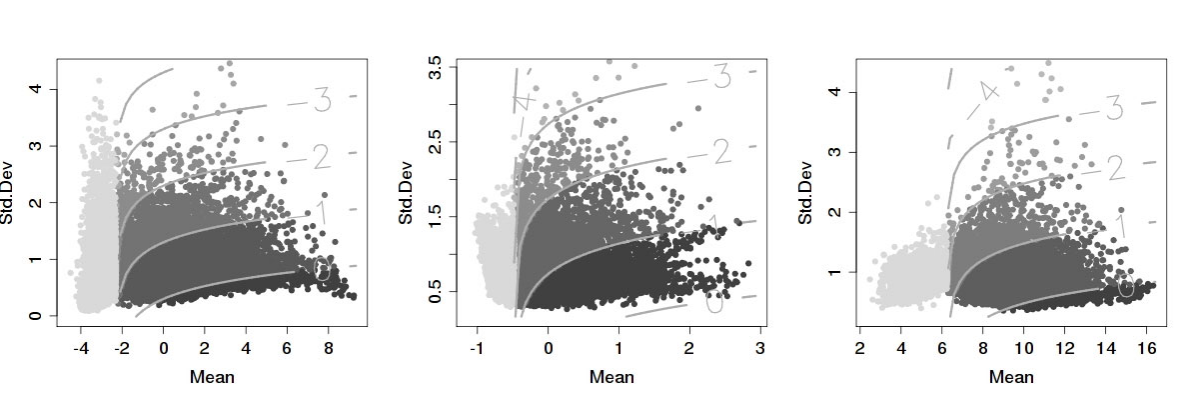
**Scatter plots of standard deviation versus mean log-intensity for BWH, NKI and EXPO-breast data sets, respectively**. The shading codes the gene stability scores, with darker colors indicating higher scores. These three data sets are from different microarray platforms. The light gray points indicate the discarded genes (those with mean expression level below the *β *value – see Eq. 1). The curves correspond to equal score levels and are one score unit apart.

These requirements lead us to propose a new *stability score *for the gene expression levels. This score for gene *i *in data set *k*, denoted *s*_*ik*_, is defined as

(1) $$s_{ik}=\alpha \log_{2}(\max\{{\hat{\mu }}_{ik}-\beta_{k},0\})-{\hat{\sigma }}_{ik},$$

where ${\hat{\mu }}_{ik}$ and ${\hat{\sigma }}_{ik}$ are the estimated mean log-intensity and the standard deviation of the gene *i *in data set *k*. The *α *coefficient allows the user to control the trade-off between the mean expression and the standard deviation in gene scoring. Results reported here were obtained with *α *= 0.25. The *β*_*k *_parameter allows one to define the level of mean expression below which the genes are not considered for ranking, *i.e*. the score for these genes is -∞. We have set *β*_*k *_to be the 25*th *percentile of the mean expression, for each data set *k*. Genes having a higher score are considered more suitable as control genes. As we see from Eq. 1, high variation in gene expression leads to a lower score when mean expression levels are equal. This is one reason we select the most highly variable probeset from the probesets representing the same gene, in order to encompass the worst-case scenario. Note also that there is no need to normalize the scores to make them comparable across data sets, because they are used solely for ranking the genes within the same data set. Finally, having computed the scores for all the genes within a data set, we order the genes from high to low values of the scores, with ties resolved by ordering by the mean expression (from high to low). From this perspective, the scores can be seen as defining classes of equivalence among genes: all the genes in the same class (having the same score) are equally useful as normalization genes. By using the second ordering criterion, we can select control genes with a desired expression level (examples of classes of equivalence are the equal score levels in Figure 2).

Figure 2 displays the influence of the mean expression level and the standard deviation on the gene score. All genes located on the curves have the same score value (they belong to the same equivalence class). Two consecutive curves are separated by one score unit.

Using this stability score, we ranked the genes from each data set, obtaining the lists that will be later combined. An excerpt from the ten lists for the breast cancer data sets is shown in Table 2 (first ten columns).

**Table 2:** Top 20 control genes from the ten breast cancer data sets and top 20 genes from the aggregated list (Meta column)

BWH	EMC	JRH2	MGH	NKI	STOCK	TGIF1	UNC	UPP	EXPO-breast	Meta
RPL37A	PPIA	RPL41	ZNF557	UBC	RPS11	RPL41	RPS10	RPL9	CALM2	RPL37A
RPL41	CALM2	RPL39	CDR1	UBB	RPS24	RPL37A	RPS18	RPL37A	HNRPA1	RPL27A
RPS18	SRP14	RPL23A	PPP1R2	OAZ1	RPL9	EEF1A1	RPLP1	ACTG1	NACA	RPS18
RPL39	RPL37A	RPL37A	TCN2	DYNLL1	RPL37A	RPL30	RPS11	RPL27A	UBA52	RPL30
RPL23A	RPS18	EEF1A1	SSBP1	RAPSN	RPL41	RPL39	RPS23	CFL1	LAPTM4A	RPL41
RPL9	RPL30	RPS23	RPL27A	PCBP1	RPL27A	PPIA	RPL37A	RPS11	RPL27A	CALM2
RPLP1	RPL27A	RPS27	RPS3	KCNH3	RPL39	ACTG1	RPL11	RPS13	RPL30	RPL27
RPS27	RPS11	CALM2	BRCC3	RPL3	RPLP1	CFL1	RPS15	RPL27	RPL9	K-ALPHA-1
RPL27A	RPL39	RPS18	PTMA	RPL8	UBB	RPS23	RPL14	RPL41	RPL31	RPS11
RPL30	RPS15	ACTG1	ABCF2	MYL6	RPS15A	RPL10	NACA	RPS18	RPL37	RPL39
RPS29	RPS24	RPL10	PCDH18	RPL14	CALM2	CALM2	RPL36AL	RPS15	RPS11	RPS13
ACTG1	RPL32	RPS24	LAX1	RPL7A	NACA	RPS11	UBA52	RPL6	RPS29	NACA
CALM2	RPS15A	RPS15A	TPMT	FAU	RPL30	HNRPA1	NEDD8	RPLP1	RPS24	RPL23A
RPS13	RPLP1	RPL32	GALE	ARF1	CFL1	RPL6	PCBP1	RPL32	RPS13	RPS24
HNRPA1	RPL9	RPL27A	MTCH1	CCT3	RPS13	RPL23A	NDUFB2	RPL31	RPS21	HNRPA1
RPS24	UBB	UBB	ATP5G2	PSAP	RPS3A	K-ALPHA-1	HNRPM	RPL39	UBB	RPL9
RPL31	K-ALPHA-1	RPS29	SF3B2	CD81	RPL37	RPS18	HNRPC	UBB	RPS27A	RPLP1
RPL34	RPS13	RPL30	SND1	SQSTM1	RPS18	EEF1G	NDUFB8	RPS24	RPS15	RPL32
RPS15A	RPL27	PPIA	RPL5	K-ALPHA-1	RPL27	TUBA6	ATP5J2	RPS27	RPL32	LAPTM4A
RPS21	FAU	CFL1	SKAP2	CALR	RPL24	RPS3A	TARDBP	DDX5	RPL24	RPS15A

#### Combining results from different data sets

Once genes are ranked according to their scores in each data set (lower ranks correspond to higher scores), the natural next step is to combine these rankings into a global ranked list. We combine the ranks of the genes rather than their scores to avoid normalizing the scores across different data sets, thereby achieving platform-independence. To this end we use the *rank product score *[16], which is a fast and efficient method for combining ranked lists. It computes, for each gene *i *∈ *G*, a new score

(2) $$R_{i}={\left(\prod_{k}{\text{rank}}_{k}(i)\right)}^{\frac{1}{n_{i}}},$$

where rank_*k*_(*i*) is the rank of *s*_*ik*_, the score for gene *i *in data set *k *(topmost gene has the rank 1), and *n*_*i *_is the number of data sets in which the gene *i *appears. The final list is obtained by sorting the genes in increasing order of *R*_*i*_. The top 20 genes from the aggregated breast cancer list are given in the 'Meta' (last) column of Table 2.

#### Validation of the aggregated lists

There is no absolute criterion by which one can judge the quality of the resulting lists. Rather, the aggregated list could be used to select from the top genes (100, for example) those genes that also satisfy further conditions of the specific application.

**Figure 3:**
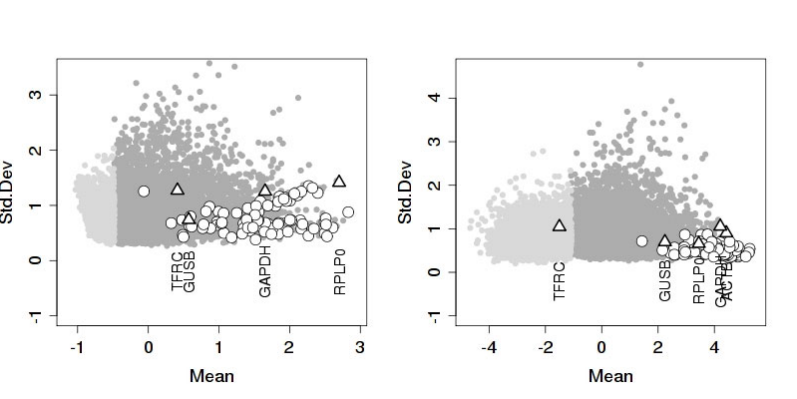
**Scatter plots of standard deviation versus mean log-intensity for two validation data sets (from left to right: NKI and UPP)**. The top 100 breast cancer control genes resulting from aggregating eight data sets are plotted as circles. Triangles correspond to the five control genes used in [17] (NKI does not contain the ACTB gene).

We can, however, have a subjective impression of the validity of the aggregated list by visualizing the resulting top genes in data sets not used for producing the list. We obtained a list of the top 100 genes by applying the method described above on eight of the ten data sets, leaving NKI and UPP aside as validation sets. The top 100 genes in both validation sets (different microarray platforms) are plotted in Figure 3. As a comparison, we also include the five control genes used in [17] (represented as triangles in the figure). It is seen that the genes are generally concentrated in the lower right part of the plot, corresponding to high mean expression levels and low variance. There is a notable difference between the quality of the results (given by the concentration of the control genes in the lower right corner) on the two platforms, due to the fact that most of the data sets used for gene selection are from Affymetrix platforms. While the top 100 lists contain genes with high stability scores on the Affymetrix platforms (the UPP data set), on the custom Agilent platform (NKI) there are a number of genes that are missed. Nevertheless, those selected still function well as control genes.

### Control genes lists

We have analyzed ten different data sets which have samples hybridized on different versions of Agilent and Affymetrix platforms. Using our proposed method, we compiled two different lists of candidate control genes: one specific to breast cancer [see Additional file 1] and one resulting from the analysis of eight different types of cancer, thus applicable to cancers in general [see Additional file 2]. From the breast cancer list we selected two new control genes which were validated in an RT-QPCR assay that also included five previously used control genes (ACTB, TFRC, GUSB, RPLP0 and GAPDH – see [17]) and breast cancer-related genes (*e.g*. ESR1, ERBB2, AURKA, *etc*.). The RT-QPCR results confirm the findings from the microarray analysis and show that more stably expressed control genes can be selected by applying the criteria mentioned above. Also, they provide empirical evidence supporting the working hypothesis that PCR control genes can be selected from microarray data.

The list of the top 50 control genes obtained from the ten breast cancer data sets is given in Table 3. More comprehensive lists, including one containing the top 2000 candidate breast cancer genes and a similar list compiled from eight different types of cancer, are available [see Additional file 1 and Additional file 2]. In the case of breast cancer control genes, it is interesting to note that some of the "classical" genes (*e.g*. ACTB, GAPDH, TFRC) are not among the top 50.

**Table 3:** Top 50 control genes as resulting from aggregating the ten breast cancer data sets. Two genes – RPS11 and UBB – were selected as control genes and validated by RT-PCR

Rank	Gene symbol	Gene ID	Description
1	RPL37A	6168	ribosomal protein L37a
2	RPL27A	6157	ribosomal protein L27a
3	RPS18	6222	ribosomal protein S18
4	RPL30	6156	ribosomal protein L30
5	RPL41	6171	ribosomal protein L41
6	CALM2	805	calmodulin 2 (phosphorylase kinase, delta)
7	RPL27	6155	ribosomal protein L27
8	K-ALPHA-1	10376	alpha tubulin
9	**RPS11**	6205	ribosomal protein S11
10	RPL39	6170	ribosomal protein L39
11	RPS13	6207	ribosomal protein S13
12	NACA	4666	nascent-polypeptide-associated complex alpha polypeptide
12	RPL23A	6147	ribosomal protein L23a
14	RPS24	6229	ribosomal protein S24
15	HNRPA1	3178	heterogeneous nuclear ribonucleoprotein A1
16	RPL9	6133	ribosomal protein L9
17	RPLP1	6176	ribosomal protein, large, P1
18	RPL32	6161	ribosomal protein L32
19	LAPTM4A	9741	lysosomal-associated protein transmembrane 4 alpha
20	RPS15A	6210	ribosomal protein S15a
21	DYNLL1	8655	dynein, light chain, LC8-type 1
22	ACTG1	71	actin, gamma 1
23	TUBA6	84790	tubulin, alpha 6
24	SRP14	6727	signal recognition particle 14kDa (homologous Alu RNA binding protein)
25	MYL6	4637	myosin, light chain 6, alkali, smooth muscle and non-muscle
26	RPL24	6152	ribosomal protein L24
27	FAU	2197	Finkel-Biskis-Reilly murine sarcoma virus (FBR-MuSV) ubiquitously expressed (fox derived); ribosomal protein S30
28	RPL31	6160	ribosomal protein L31
29	RPS15	6209	ribosomal protein S15
30	MTCH1	23787	mitochondrial carrier homolog 1 (C. elegans)
31	**UBB**	7314	ubiquitin B
32	RPL37	6167	ribosomal protein L37
33	HMGN2	3151	high-mobility group nucleosomal binding domain 2
34	RPS27	6232	ribosomal protein S27 (metallopanstimulin 1)
35	GDF8	2660	growth differentiation factor 8
36	RPL38	6169	ribosomal protein L38
37	RPS29	6235	ribosomal protein S29
38	SULT1C2	27233	sulfotransferase family, cytosolic, 1C, member 2
39	RPL6	6128	ribosomal protein L6
40	UBC	7316	ubiquitin C
41	UBA52	7311	ubiquitin A-52 residue ribosomal protein fusion product 1
42	MRFAP1	93621	Mof4 family associated protein 1
43	HNRPK	3190	heterogeneous nuclear ribonucleoprotein K
44	PARK7	11315	Parkinson disease (autosomal recessive, early onset) 7
45	PSMC1	5700	proteasome (prosome, macropain) 26S subunit, ATPase, 1
46	LOC158572	158572	hypothetical protein LOC158572
47	RPS8	6202	ribosomal protein S8
48	ATP5A1	498	ATP synthase, H+ transporting, mitochondrial F1 complex, alpha subunit 1, cardiac muscle
49	EIF4H	7458	eukaryotic translation initiation factor 4H
50	CD63	967	CD63 molecule

### Evaluation of control genes by RT-QPCR

**Figure 4:**
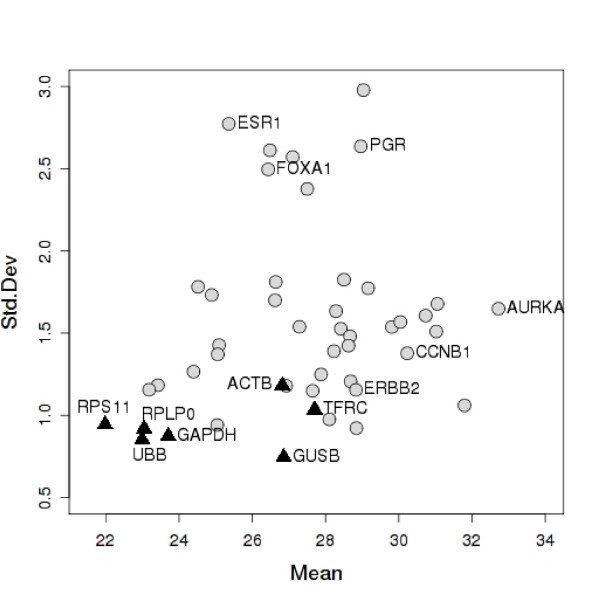
**RT-QPCR experiment**. Standard deviation as a function of the mean expression level (expressed as raw Ct values) of 47 genes in a RT-QPCR experiment. Higher expression levels correspond to smaller raw Ct values. Control genes are represented by triangles, test genes by circles. The new control genes RPS11 and UBB are in the lower left corner.

Motivated by the consistency of the selection process for suitable control genes among different microarray platforms, we performed a small scale RT-QPCR experiment to test the performance of two new control genes along with a number of more commonly used control genes. In this experiment, RNA was isolated from 25 cryo-preserved breast cancer samples and the expression of 47 genes was measured by RT-QPCR [18]. Test genes were selected according to their relatedness to proliferation or estrogen receptor functions. Some of the test genes had been previously identified and used for characterizing primary breast cancers [17]. Two genes, RPS11 and UBB, ranked 9 and 31 in Table 3 respectively, were compared to five additional control genes and to a number of test genes previously measured by [17]. Mean raw expression values of all candidate control and test genes were plotted against standard deviations of each gene (Figure 4). The raw Ct (cycle threshold) value is the number of PCR cycles required for the fluorescence signal to cross the background threshold, so that low Ct values correspond to high expression levels. RPS11 and UBB are clearly among the most stably expressed genes, as their standard deviations are both quite low. Other genes frequently used as control genes are also shown. For comparison, mean expression and standard deviation of several test genes are also indicated. The expression of most test genes is much more variable than UBB and RPS11.

The two new control genes, together with RPLP0, offer the best trade-off between mean expression level and variability, while others like ACTB or TFRC are less stably expressed and therefore seem less suitable for use as normalization genes.

## Discussion

We propose a new approach which leverages publicly available microarray data to produce lists of candidate control genes for RT-QPCR. Our method is independent of the microarray platform or normalization methodology, and is able to cope with gene lists that overlap only partially. After screening thousands of genes (generally more than 10,000 genes in each data set), we have produced two separate lists of candidate genes: one specific to breast cancer and one generally applicable to different types of cancer. We do not consider these lists as generally applicable, as the data used do not allow such generalization. Different pathologies may have a different impact on the control genes and some of the control genes we selected may become ineffective in the case of a disease which affects their particular functions. On the other hand, more diverse data should be used if the goal is finding global control genes. The list of the top 50 breast cancer control genes (Table 3) is dominated by ribosomal proteins. This finding is consistent with the fact that ribosomes are a major component of basic physiologic processes in all the cells and not a primary target of changing conditions. Other genes present among the first 50 genes code for protein turnover (ubiquitin), tubulin-related proteins or actins, structures which are required in all living cells.

Our results are supported by recent findings of de Jonge *et al*. [5], who used a different ranking method. In addition, the lists of control gene candidates for breast cancer and for diverse types of cancer are similar [see Additional file 1 and Additional file 2], as a large number of the top ranked genes belong to the same functional category (ribosomal genes, protein turnover).

Another important finding is that some of the commonly used control genes in breast cancer (ACTB and TFRC) appear to be less stable than previously assumed. This has an impact on the normalization strategy of the QPCR measurements: indeed, in our more recent experiments we have chosen to use the mean of RPLP0, RPS11 and UBB (on the log_2 _scale) for normalizing the expression of test genes.

Finally, we would like to emphasize that these two lists should not be taken in an absolute sense: a gene in top 10 is not necessarily a better choice than a gene in the top 20 to 30. But we do consider it to be definitely a better candidate than a gene not in top 100. Nor do we consider the resulting ranking as providing a solution to the problem of finding normalization genes in all contexts. Rather, the lists produced through this process are meant to guide the choice of control genes while also taking into consideration the specific requirements of any individual analysis. Depending on the planned application, other parameters must be considered. For example, short amplicons or intron-spanning primers must be used when the starting RNA is considerably degraded or when residual DNA contaminations might affect QPCR. The final choice of control genes should be made not by blind adherence to the ranked list, but be imposed by the intended application.

## Conclusion

Starting from clearly defined criteria, we have designed a novel method for ranking the candidate genes for their suitability as control genes in RT-QPCR experiments. The genes from a data set were ranked according to their stability score, which represented a trade-off between gene's average expression level and its variance. Finally, the rankings from several data sets were combined into a list of candidate genes, with higher ranked genes being considered to be more suitable as control genes. The proposed approach had the advantage of being platform- and normalization- independent and of not being restricted to only the list of common genes across all data sets.

By applying the proposed method to two particular collections of data sets we were able to produce two lists of candidate genes from which control genes for either breast cancer or more diverse cancer could be easily selected. Two new control genes for breast cancer – UBB and RPS11 – have been identified and validated by RT-QPCR.

Our results support the hypothesis that selecting control genes for QPCR from microarray data is feasible.

## Authors' contributions

VP conducted the analysis, devised algorithms and wrote the computer programs. PW collected the datasets and remapped the probes. PW, DRG and MD designed the study and statistical analyses. JA and RJ initiated the biological problems and conducted the RT-PCR validation. All authors have read and approved the final manuscript.

## Supplementary Material

Additional file 1 **Top 2000 breast cancer candidate control genes.** Excel file containing top 2000 genes as resulted from combining the ten breast cancer data sets. Click here for file

Additional file 2 **Top 2000 diverse cancer candidate control genes.** Excel file containing top 2000 genes as resulted from combining the eight different types of cancer from the EXPO data set. Click here for file
